# A preliminary study of the novel antibiotic-loaded cement computer-aided design-articulating spacer for the treatment of periprosthetic knee infection

**DOI:** 10.1186/s13018-019-1175-0

**Published:** 2019-05-16

**Authors:** Chun-Hao Tsai, Horng-Chaung Hsu, Hui-Yi Chen, Yi-Chin Fong, Mao-Wang Ho, Chia-Huei Chou, Yi-Wen Chen, Ming-You Shie, Tsung-Li Lin

**Affiliations:** 1 Department of Orthopedics, China Medical University Hospital, No. 2, Yude Road, Taichung, 40447 Taiwan; 2 School of Medicine, China Medical University, Taichung, 40447 Taiwan; 3 Department of Sports Medicine, China Medical University, Taichung, 40447 Taiwan; 4 Graduate Institute of Biomedical Sciences, China Medical University, Taichung, 40447 Taiwan; 5 Department of Radiology, China Medical University Hospital, Taichung, 40447 Taiwan; 6 China Medical University, Beigang Hospital, Yunlin, 40447 Taiwan; 7 Division of Infection Disease, Department of Internal Medicine, China Medical University Hospital, Taichung, 40447 Taiwan; 8 3D Printing Medical Research Center, China Medical University Hospital, Taichung, 40447 Taiwan

**Keywords:** Computer-aided design, Articulating spacers, Periprosthetic knee infection, Two-staged revision, Mechanical complications

## Abstract

**Background:**

In comparison to static spacers, articulating spacers have been shown to result in a similar infection eradication rate in two-stage revision of periprosthetic knee infections. However, the optimal construct for articulating spacers has not been identified yet. The aim of this study was to present a preliminary result of treatment for periprosthetic knee infection using a novel computer-aided design (CAD)-articulating spacer.

**Methods:**

We retrospectively reviewed 32 consecutive cases of chronic periprosthetic knee infection occurring from January 2015 to December 2015. In these cases, we used an antibiotic-loaded, optimized CAD-articulating spacer based on the retrieved knee prosthesis. Evaluation included infection eradication rate, the Hospital of Special Surgery (HSS) knee score, range of motion (ROM), and spacer-related mechanical complications. All cases were regularly followed-up for 2 years minimum.

**Results:**

Twenty-eight of 32 patients (87.5%) had infection eradication; 18 patients (56.3%) received reimplantation successfully. The mean interval between spacer insertion and reimplantation was 8.8 months (range 4.0–12.5 months). The mean HSS knee score and ROM significantly increased during each interval (*p* < 0.0001 for both). The mean HSS knee scores were 31.2 (range 20–48) at initial visit, 65.4 (range 60–78.8) at 1 month after spacer insertion, and 84.2 (range 78–90) at 3 months after reimplantation (*p* < 0.0001). The mean ROM were 72.0° (range 15–100°), 85.6° (range 35–110°), and 102.0° (range 80–122°), respectively (*p* = 0.002). Two (6.3%) spacer-related mechanical complications occurred.

**Conclusions:**

The CAD-articulating spacer in two-staged revision of periprosthetic knee infection significantly controlled infection, improved clinical outcomes, increased ROM, and decreased mechanical complications in the preliminary study. Further larger clinical studies are needed to confirm the findings presented here.

## Background

Chronic periprosthetic infection is a devastating complication following total knee arthroplasty (TKA), and two-stage revision with an antibiotic-loaded spacer is the gold standard for treatment due to high infection eradication rates (as high as 80–90%) [1–4]. In comparison to static spacers, articulating spacers have been shown to result in a similar infection eradication rate. However, articulating spacers may provide better ROM and mobility, improved functional outcome, some weight-bearing, and easier subsequent reimplantation [4]. Although there are several types of articulating spacers, such as cement-on-cement handmade (COCH), cement-on-cement prefabricated (COCP), cement-on-cement molded (COCM), and metal-on-polyethylene (MOP), the optimal articulating spacer construct has not yet been identified [5]. Moreover, several articulating spacer-related mechanical complications have occurred, such as extensor apparatus problems, spacer loosening or fracture, and joint subluxation or dislocation; as many as 57% of cases have reported these complications [5, 6]. These problems might lead to further surgery, poor functional outcomes, prolonged treatment courses during interim stage, and difficulty in reimplantation [7, 8].

In the last decade three-dimensional (3D) printing technology has been applied more frequently in orthopedic surgical techniques [9], such as reverse engineering [10], computer-aided design (CAD) [11], computer-aided manufacture (CAM) [12], and rapid prototyping [13]. It was speculated that the CAD technique may help optimize articulating spacers [5].

Therefore, COCM articulating spacers were designed and fabricated using the CAD technique and were based on the retrieved knee prosthesis. This preliminary study was conducted to evaluate these CAD-articulating spacers and their efficacy in treating periprosthetic infection following TKA two-stage revision. It was hypothesized that these optimized spacers could (1) eradicate periprosthetic knee infection, (2) improve clinical outcomes, (3) increase ROM, and (4) decrease mechanical complications.

## Methods

### Design of CAD-articulating spacers

First, 3D computer models with reverse engineering were obtained. The smallest size of the right side retrieved knee prosthesis (U2 PSA Revision Knee; United Orthopedic Corporation, New Taipei City, Taiwan) was chosen as a prototype. The femoral and tibial insert components were scanned by the SmartSCAN-HE 3D scanner (Accurex, Braunschweig, Germany). The data were imported into Geomagic Design X (3D Systems Corporation, South Carolina, USA) to create 3D virtual models. Six sizes of the 3D femoral and tibial insert models were available after proportional amplification of the prototype (amplification was done without changing the curvature and geometry of the articular surface in Geomagic Design X).

**Fig. 1:**
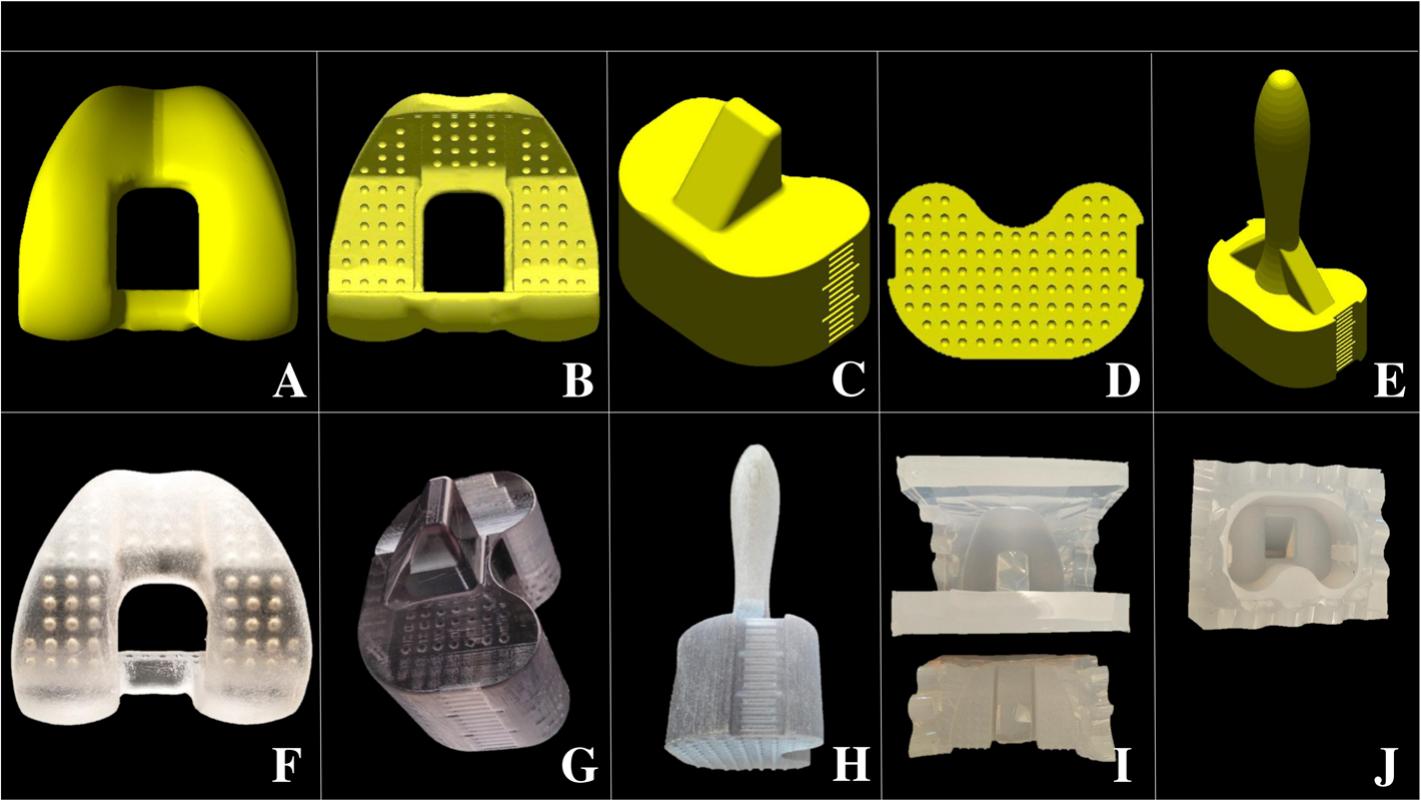
In the femoral model, **a** the view from articular surface showed the centrally drawn trochlea groove; **b** the view from the bony surface showed multiple 2.0 mm in diameter with 4.0 mm separating the concave semicircles in a checkerboard pattern; in the tibial insert model, **c** the view from the articular surface showed 30.0-mm thickness, with 2.0-mm mark intervals on bilateral surfaces as a scale; **d** the view from the bony surface showed multiple convex semicircles with the same pattern as in the femoral models; **e** the tibial molder based on the tibial insert model with a handle; **f**, **g**, and **h** fabrication of the CAD-femoral and CAD-tibial trials and tibial molder; **i** and **j** each corresponding silicone mold for fabricating the femoral and tibial cement spacers

Next, the articulating spacer was optimized with the CAD technique. In each femoral model, the trochlea groove was drawn centrally (Fig. 1a). The femoral cam and sagittal box were preserved, but the femoral stem and axial box were removed. All the depressions, gaps, or defected areas of the femoral models were drawn, filled, and aligned with the edge of the plane to smoothen the contour. Moreover, multiple concave semicircles that were 2.0 mm in diameter were created over the bony surface of femoral models in a checkerboard pattern, with 4.0 mm separating each semicircle (Fig. 1b).

In each tibial insert model, the post was preserved. The thickness of the insert was enlarged to 30.0 mm, with 2.0-mm intervals marked on the medial and lateral surfaces of the model to be used as a scale (Fig. 1c). The same procedure was done in femoral models to fill all defect areas. Conversely, multiple convex semicircles, with a 2.0-mm diameter, were created over the bony surface of tibial insert models with the same spread pattern as in femoral models (Fig. 1d). Each “tibial molder” was designed based on the tibial insert model, with removal of the post and attachment of a “handle” (Fig. 1e).

The last step (CAM) was to fabricate the femoral and tibial insert models as CAD-femoral and CAD-tibial trials using the Objet500 Connex3 3D printer (Stratasys, USA) using biocompatible resin MED610 (EN-International Standards Organization [ISO] 10993-5:2009, 10993-10:2013, 10993-3:2014, 10993-18:2009, USP Plastic Class VI USP 34 <88>) (Fig. 1f, g). Each tibial molder was manufactured with the same method (Fig. 1h). The corresponding molds for each CAD-femoral and CAD-tibial trials were manufactured with medical grade silicone rubber TSE3488T (Momentive, Gunma, Japan) (Fig. 1i, j). The silicone molds were made of a thermosetting polymer, and could be resterilized by hydrogen peroxide plasma for repetitive use without shape deformation.

### Patient selection

This study was approved by the Institutional Review Board (CMUH107-REC3-130) and was designed as a series of retrospective consecutive cases. Adult patients with chronic periprosthetic knee infections after primary TKA were enrolled between January 2015 and December 2015. They underwent two-stage revision with the insertion of the CAD-articulating spacer and were followed up for a minimum of 2 years. All cases met the Musculoskeletal Infection Society (MSIS) criteria [14], and all exhibited chronic infection with a clinical duration lasting longer than 1 month [2]. Loosening of components was diagnosed by plain radiography. All surgeries were performed by a single fellowship-trained arthroplasty surgeon (TLL).

### Surgical technique

Patients were given spinal anesthesia and placed in the supine position. The medial parapatellar approach was performed for all knees, with the use of a tourniquet. During the debridement, multiple soft tissues and fluid specimens were obtained, removed, and sent for culture. All the components, cement, synovium, and necrotic tissue were removed thoroughly, and care was taken to avoid fracture.

**Fig. 2:**
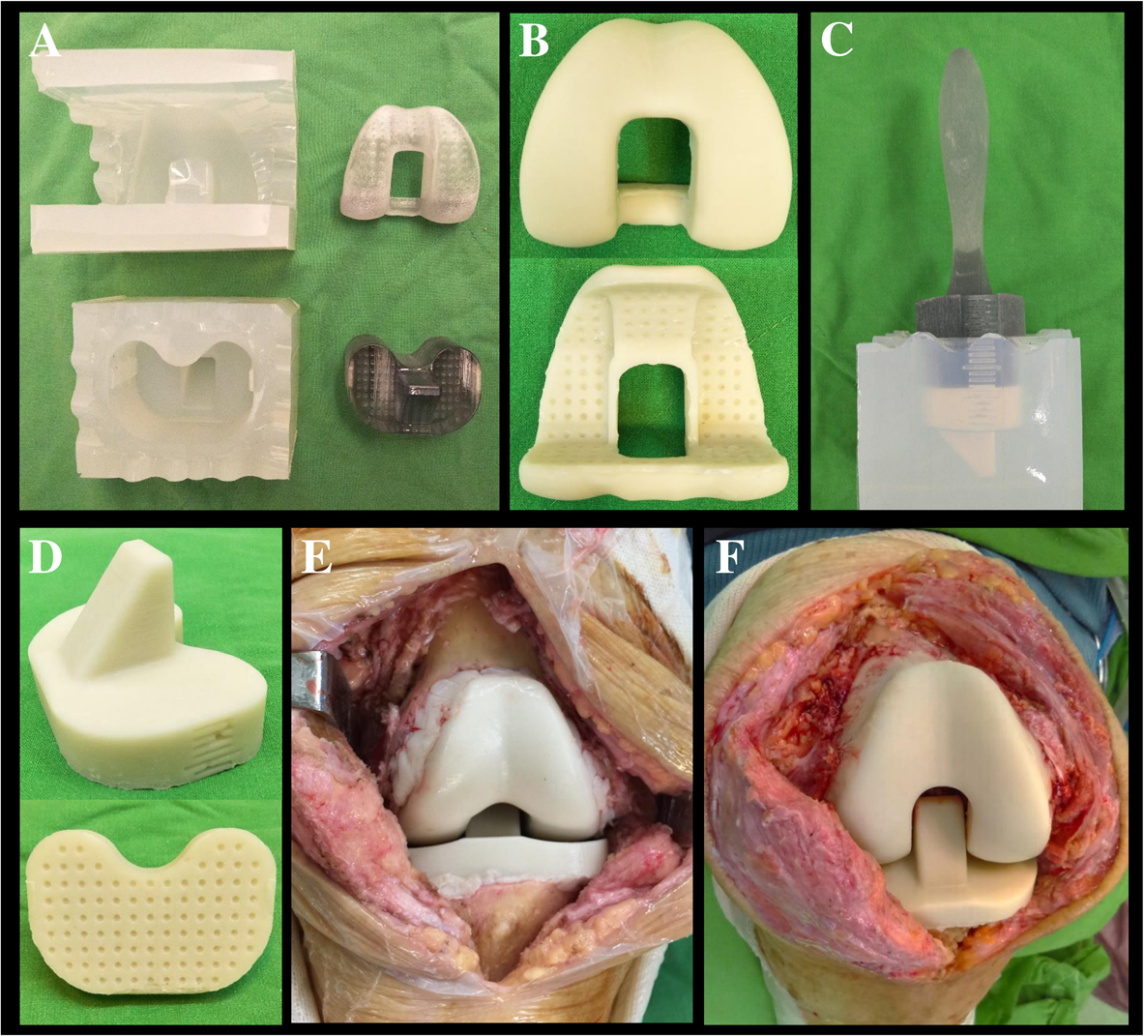
**a** The corresponding silicone molds were chosen after sizing against the retrieved prosthesis; **b** antibiotic-loaded cement CAD-femoral spacer; **c** the adequate thickness of the tibial spacer was determined with the silicone mold and tibial molder; **d** antibiotic-loaded cement CAD-tibial spacer; **e** and **f** intraoperative photograph of the assessment of stability and tracking of the knee joint after insertion of CAD-articulating spacers

After component removals, molding of the CAD-femoral and CAD-tibial spacers were performed by another surgeon. Each of these two steps took approximately 30 min. The debridement was performed by one team of surgeons at the same time and took about 60 min. The CAD-femoral and CAD-tibial trials were sized against the ones retrieved. Then, the corresponding silicone molds were chosen for fabricating the cement spacer (Fig. 2a). The femoral spacer was prepared first. Antibiotics with 4.0 g vancomycin and 4.0 g ceftazidime were mixed into one package of CMW3 bone cement (DePuy Synthes, Warsaw, IN, USA) [15, 16]. The use of lubricant was not necessary because the cement did not adhere to the interface of the silicone mold. After the cement cured, the femoral spacer was easily separated from the mold (Fig. 2b). An additional package of antibiotic-loaded cement (comprised of the same formula) was used to implant the spacer in the distal femur. Gelfoam was packed in the metadiaphyseal junction to avoid infiltration of cement, but to fill the metaphyseal defect with stem-like cement. The cement technique was applied early to the spacer and late to the open bone ends, to allow molding to the bony defect surface without adherence and interdigitation [3].

After the cement had cured, adequate gap tension was measured under traction of the leg at full extension and the flexion of the knee joint was 90°; this was determined by using the depth scale on the CAD-tibial trial. The thickness of the tibial spacer was determined and prepared with the aid of the internal depth gage of the tibial mold and molder (Fig. 2c). Then the tibial spacer was easily separated from the mold after curing of cement (Fig. 2d). The same cement techniques were used for implanting the tibial spacer in the proximal tibia. The limb was taken to full extension in correct alignment and tension, allowing the tibial spacer to seek its appropriate level (Fig. 2e).

An assessment for stability and tracking was performed (Fig. 2f). Lateral release was performed if necessary. Then the wound was closed in layers with monofilament sutures and closed suction drains that were left in situ following surgery. Any sinus tract over the skin was excised and closed.

The mean surgical time took 103.3 min (range 86–124 min). The overall surgical time was not extended because molding of the CAD-articulating spacers and debridement were performed simultaneously.

### Postoperative care

**Fig. 3:**
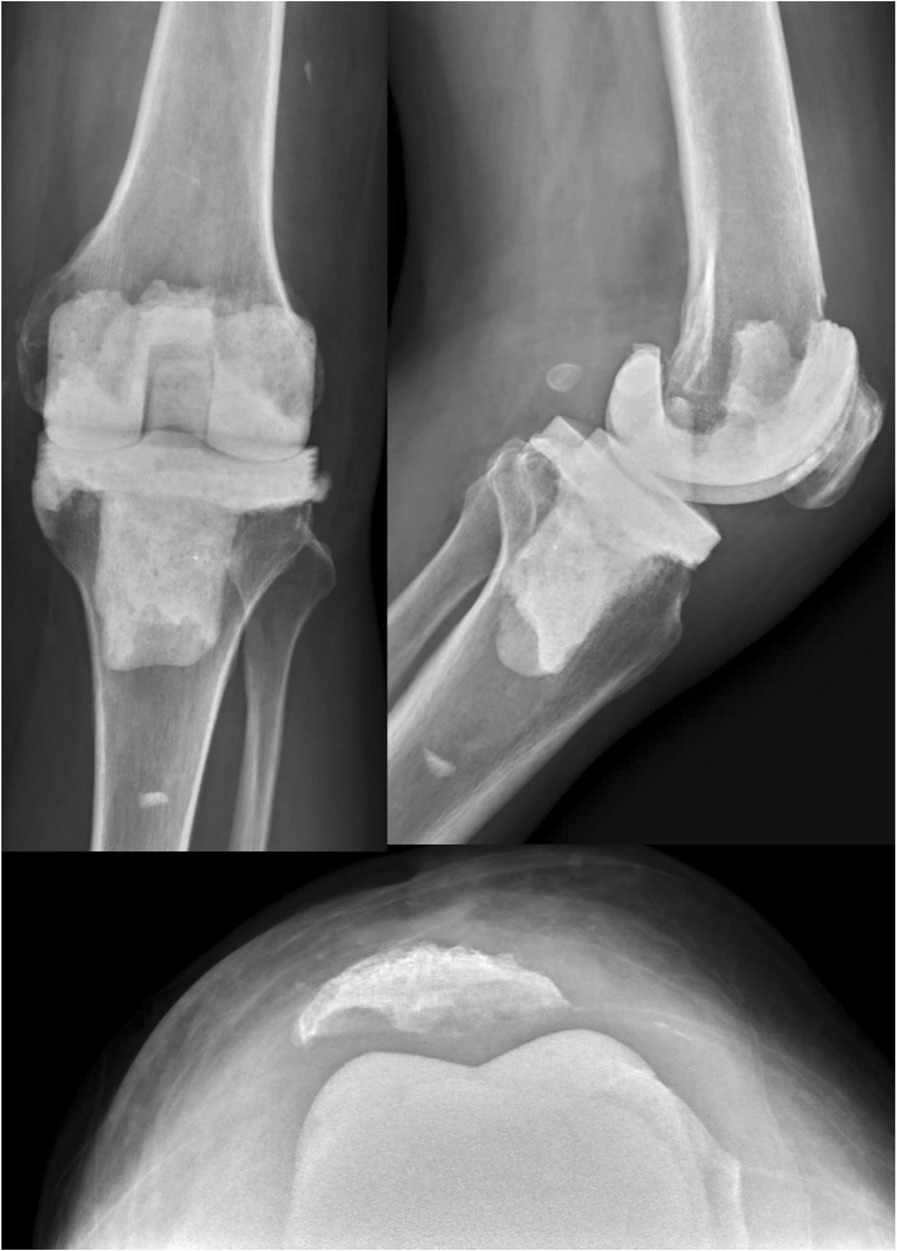
Knee anteroposterior, lateral, and skyline Merchant radiographs were taken with the CAD-articulating spacer in situ

Use of continuous passive motion device started two days following the operation; toe-touch weight-bearing (with crutch) was allowed on the third day following surgery. Full weight-bearing was not allowed during the entire interim period. A knee brace was not used. The drains were removed when the volume of fluid drainage from the surgery site was at a minimum at 1 or 2 weeks after surgery. Intravenous (IV) antibiotics were administered according to the susceptibilities of each microorganism, while intravenous vancomycin with ceftazidime was administered under culture-negative conditions [15, 16]. IV antibiotics were administered for at least 4 weeks, until a progressive decline was seen in C-reactive protein (CRP) levels and erythrocyte sedimentation rate (ESR) and the wound was healed. After discharge, oral antibiotics were continued for at least 4 weeks for suppression until CRP levels and ESR returned to normal. Radiographic evaluations were conducted immediately after the operation, 1 month postoperatively, and every subsequent month in the outpatient clinic (Fig. 3). Criteria for reimplantation included negative clinical signs of infection, CRP and ESR values within normal limits, and negative-culture of arthrocentesis after holding oral antibiotics for 2 weeks [17].

### Evaluations

As the clinical outcome evaluation, the Hospital of Special Surgery (HSS) knee score [18] and ROM were documented at the initial visit, 1 month after spacer insertion and 3 months after reimplantation. Infection eradication was defined using the Delphi criteria [17]. Radiographic evaluations on following plain films were performed in the INFINITT’s Picture Archiving and Communications System. The following codes were for recording spacer-related mechanical complications: (1) optimal size and position of the spacer, (2) spacer loosening, tilting or migration, (3) spacer crack or fracture, (4) joint subluxation (including mediolateral or anterolateral translation in relation to the spacer component), (5) joint dislocation, and (6) extensor apparatus problems (patellar maltracking or fracture) [5, 6]. One musculoskeletal radiologist (HYC) and two arthroplasty surgeons (YCF and HCH) assessed and recorded all radiographic data independently.

### Statistical analyses

Statistical analyses were performed using SPSS for Windows, version 24 (SPSS Inc, Armonk, NY). The Friedman two-way analysis of variance (ANOVA) by ranks test was used as a non-parametric test. For comparison, between each time interval, Wilcoxon signed-rank test was used. The reliability of spacer-related radiographic coding was examined by the intra-class correlation coefficient (ICC). Statistical significance was set at *p* < 0.05.

## Results

Thirty-two adult patients were enrolled. There were 14 males and 18 females, with a mean age of 73.3 years old (range 58–93 years). Twenty-one patients (65.6%) underwent debridement procedures in other institutions before visiting our clinic. All patients exhibited primary TKA, including 10 (31.3%) of cruciate retaining (CR) type and 22 (68.7%) of cruciate substituting (CS) type. The average hospital stay was 5.4 weeks (range 4–8 weeks), whereas the average duration of follow-up was 36.9 months (range 30.1–45 months). The causative microorganisms were isolated in 27 knees, with polymicrobial infection occurring in three of 27 cases; the causative agent of infection was unknown in five knees. The clinical data are presented in Table 1.

**Table 1:** Patient clinical data

Case no.	Age (year)/sex	Initial visit		Causative agent(s)	Spacer		Interval (month)	Post-reimplantation		Result	Infection eradication	Follow-up (month)
		Knee score	ROM (degree)		Knee score	ROM (degree)		Knee score	ROM (degree)			
1	67/M	34	10–80	MRSA	66	5–95	5.5	83.6	0–100	Reimplantation	Y	45
2	63/M	46.6	5–20	MSSA	68	10–45	4	86.3	5–85	Reimplantation	Y	44.8
3	67/F	26	5–95	MSSA	78.8	5–90	4.5	88.2	0–103	Reimplantation	Y	44
4	84/F	22	20–60	Unknown	68	12–85				Spacer	Y	43.5
5	74/F	32.3	3–103	*S. epidermidis*	62	5–100	4.5	90	3–105	Reimplantation	Y	42.3
6	73/F	36	5–95	*S. dysgalactiae*	60.2	0–100	8.5	85.2	0–108	Reimplantation	Y	41.8
7	68/M	32	3–97	*C. koseri*	69.3	5–100	2*			Resection	N	40.8
8	88/F	38	10–87	MSSA	66.2	6–95				Spacer	Y	40.2
9	62/M	28	12–83	MSSA	64	2–90	4.5	88.3	0–94	Reimplantation	Y	39.6
10	71/M	42.8	6–100	Unknown	67	10–120	5.5	80.2	3–125	Reimplantation	Y	39.2
11	77/F	38	5–90	MSSA	66.5	6–116	6	78	2–120	Reimplantation	Y	38.5
12	68/M	20	30–50	MRSA + *P. aeruginosa*	60	20–68	3.5	81.2	5–85	Reimplantation	Y	38.2
13	71/F	36.7	12–85	*E. coli*	70.2	5–108	5.5	83.2	0–112	Reimplantation	Y	38
14	58/F	42	5–90	Unknown	62.2	10–95	6	86	5–103	Reimplantation	Y	37.8
15	93/M	20	70–85	*E. cloacae*	64	45–95				Spacer	Y	37.3
16	85/M	22	13–92	*S. haemilyticus*	63.5	10–90				Spacer	Y	37.1
17	72/M	22.2	8–90	Unknown	67	10–100	1.5*			Resection	N	36.7
18	70/F	34	5–96	*S. salivarius*	65	8–100	5	82.1	5–108	Reimplantation	Y	36.4
19	65/F	24	30–80	*C. striatum*	61	6–90	4.5	83.6	2–95	Reimplantation	Y	36.2
20	63/M	26.6	13–85	*P. aeruginosa*	60.8	10–95	2.5*			Resection	N	35.6
21	66/F	32	3–95	VRE	61.2	0–110	12.5	80.4	0–115	Reimplantation	Y	34.4
22	86/M	42	5–93	*E. coli*	64.5	5–103				Spacer	Y	34
23	71/M	20.1	20–75	MRSA + *E. cloacae*	68.6	20–77	3.8*			Amputation	N	33.8
24	76/F	23	3–92	MRSA	64	6–100				Spacer	Y	33.5
25	78/M	33	10–90	*P. aeruginosa*	63	8–96				Spacer	Y	33.5
26	62/F	39	5–85	*E. cloacae*	67.5	5–95	6.3	82.3	0–108	Reimplantation	Y	32.4
27	81/F	36	10–85	*E. coli*	66	5–102				Spacer	Y	32.3
28	89/M	23	6–85	Unknown	69.4	4–96				Spacer	Y	32
29	85/F	20	30–65	MSSA + *S. dysgalactiae*	61	25–85				Spacer	Y	31.8
30	70/M	48	5–93	MSSA	68	5–100	4.2	84.2	3–110	Reimplantation	Y	31.4
31	72/F	30.5	6–84	MRSA	64	3–88	4.5	83.6	0–95	Reimplantation	Y	30.8
32	63/F	27.7	12–86	MSSA	67.2	10–90	6.4	89.6	4–102	Reimplantation	Y	30.1
Average	70.4	31.2	72.0		65.4	85.6	8.8	84.2	102.0			36.9

*M*, male; *F*, female; *ROM*, range of motion; *MRSA*, methicillin-resistant *Staphylococcus aureus*; *MSSA*, methicillin-sensitive *Staphylococcus aureus*; *S. epidermidis*, *Staphylococcus epidermidis*; *S. dysgalactiae*, *Streptococcus dysgalactiae*; *C. koseri*, *Citrobacter koseri*; *P. aeruginosa*, *Pseudomonas aeruginosa*; *E. coli*, *Escherichia coli*; *E. cloacae*, *Enterobacter cloacae*; *S. haemolyticus*, *Staphylococcus haemolyticus*; *S. salivarius*, *Streptococcus salivarius*; *C. striatum*, *Corynebacterium striatum*; *VRE*, vancomycin-resistant *Enterococci*; *Y*, yes; *N*, no

*Four cases with reinfection were ruled out for calculation of the average interim period

The overall infection eradication rate was 87.5% (28/32). Reinfection occurred in four knees (12.5%), which satisfied either ≥ 1 major or ≥ 3 of 5 minor MSIS criteria [14]. Of the four knees, three received resection again with CAD-articulating spacer implantation and one received amputation above the knee. There was no soft tissue defect of the knee in this series; therefore, the flap reconstruction procedure was not used. The mean time interval between spacer insertion and reimplantation was 8.8 months (range 4.0–12.5 months). Eighteen knees (56.3%) received reimplantation with NexGen Legacy Constrained Condylar Knees (LCCK) (Zimmer, Warsaw, IN, USA). At the latest follow-up, none of them had evidence of infection nor required chronic antibiotic therapy.

Ten patients (31.2%) underwent spacer retention in situ because they were medically unfit for further surgery. However, until the last follow-up for 2 years minimum, there was no recurrent infection in patients who underwent spacer retention.

The mean HSS knee scores were 31.2 (range 20–48) at initial visit, 65.4 (range 60–78.8) at 1 month after spacer insertion, and 84.2 (range 78–90) at 3 months after reimplantation (*p* < 0.0001). The mean HSS knee score significantly increased during each interval (*p* < 0.0001 between the initial visit and the first month after spacer insertion and *p* < 0.0001 between the spacer and 3 months following reimplantation). The mean ROM values were 72.0° (range 15–100°) at initial visit, 85.6° (range 35–110°) at 1 month after spacer insertion, and 102.0° (range 80–122°) at 3 months after reimplantation (*p* = 0.002). The mean ROM value significantly increased during each time interval (*p* < 0.0001 and *p* < 0.0001, respectively).

**Fig. 4:**
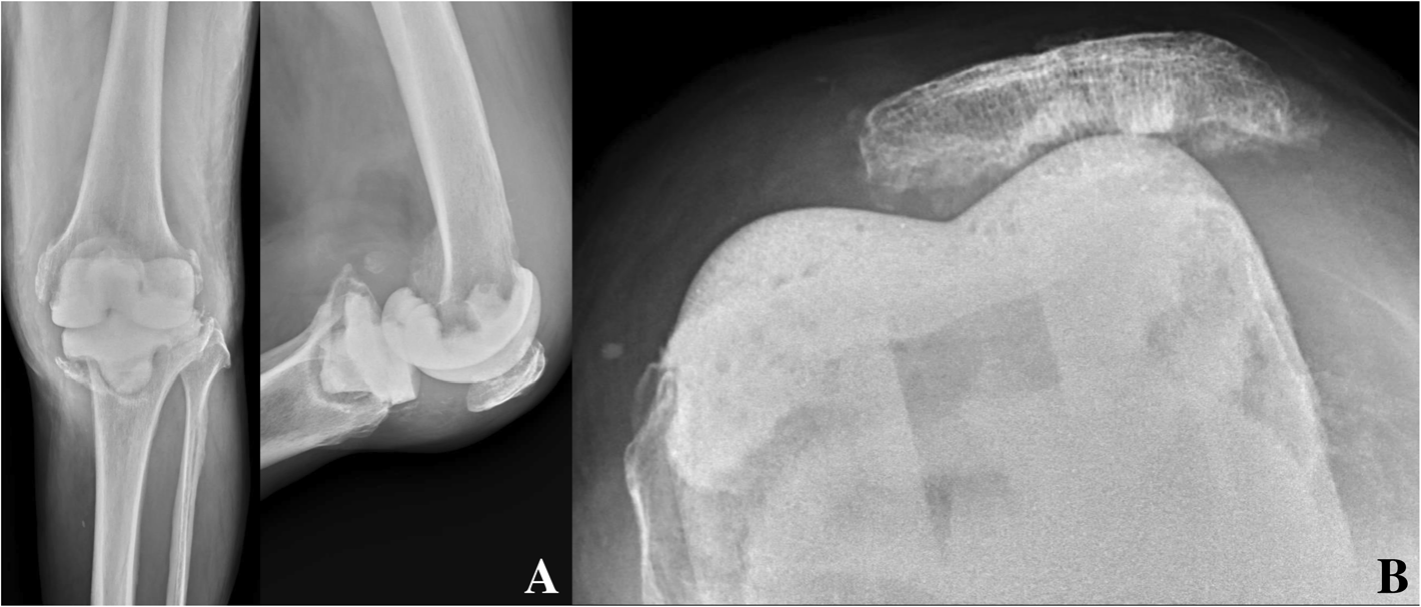
**a** Knee anteroposterior and lateral radiographs showing the medial plateau fracture with tibial spacer tilting 3 months after spacer insertion; **b** knee skyline Merchant radiograph showing patellar maltracking disorder 1 month after spacer insertion

There were two spacer-related mechanical complications, with an overall complication rate of 6.3% (2 out of 32 cases). The ICC of spacer-related radiographic coding was 0.995 (range 0.991–0.997, *p* < 0.0001) (Table 2). The first complication, in case 2, was a medial plateau fracture with tibial spacer tilting after 3 months of spacer insertion (Fig. 4a). Fortunately, the patient received fair reimplantation. The second complication, in case 13, was patellar maltracking disorder, and it occurred during the first month after spacer insertion (Fig. 4b). The patient complained of lateral knee bulging with deep flexing, but flexing did not affect ROM of the knee joint (5–108°), and the HSS knee score was 70.2. The patient received reimplantation after 5.5 months, without further complications.

**Table 2:** Mechanical complications of CAD-articulating spacers

Case no.	Spacer-related radiographic code*		
	Physician 1	Physician 2	Physician 3
1	A	A	A
2	B	C	B
3	A	A	A
4	A	A	A
5	A	A	A
6	A	A	A
7	A	A	A
8	A	A	A
9	A	A	A
10	A	A	A
11	A	A	A
12	A	A	A
13	F	F	F
14	A	A	A
15	A	A	A
16	A	A	A
17	A	A	A
18	A	A	A
19	A	A	A
20	A	A	A
21	A	A	A
22	A	A	A
23	A	A	A
24	A	A	A
25	A	A	A
26	A	A	A
27	A	A	A
28	A	A	A
29	A	A	A
30	A	A	A
31	A	A	A
32	A	A	A

*A, optimal size and position of the spacer; B, spacer loosening, tilting or migration; C, spacer crack or fracture; D, joint subluxation; E, joint dislocation; F, extensor apparatus problems

## Discussion

This was the first preliminary study to fabricate optimized articulating spacers based on retrieved knee prosthesis with reverse engineering, using CAD and CAM techniques. Particularly, it was the first study of its type to investigate the role of CAD-articulating spacers in the treatment of periprosthetic knee infection. The preliminary result demonstrated that CAD-articulating spacers in two-staged revision of periprosthetic knee infection could eradicate infection, improve clinical outcomes, increase ROM, and decrease mechanical complications.

After reverse engineering, the retrieved femur and tibial insert components, the real-sized prosthesis-like 3D models with corresponding geometry and high joint congruence prototype could be obtained. The prototype then was amplified proportionally without changing the curvature and geometry of the articular surface to obtain six different sizes of femoral and tibial insert models, respectively. With six sizes of CAD-femoral and CAD-tibial trials available, the surgeon could compare the retrieved components to select the adequate silicon mold quickly, reducing the disadvantage of the limited options regarding spacer sizes [5, 19–22]. In the present study, the size of each retrieved component was roughly compatible with CAD-trials regardless of different prosthesis brands.

Comparing using commercial prostheses as models in fabricating silicone molds, the reverse engineering and CAD techniques used in CAD-articulating spacer demonstrated the following advantages: (1) In CAD-femoral models, centralization of the trochlea area could reduce the number of instruments required for molding (for both knees) [7, 20, 23, 24]. Patellar tracking could be adjusted intraoperatively by lateral releasing; (2) the CAD-tibial trials with 2.0-mm gap and a total of 15 interval marks facilitated simple measurement of the flexion-extension gaps. The customized thickness of the CAD-tibial spacer could be molded by the internal depth gauze of the tibial mold, which was similar with StageOne knee spacers (Biomet, Warsaw, IN, USA) [6, 20, 25]; (3) the design of the novel CAD-tibial molder allowed surgeons to push the cement to make a flat plane and multiple small pits of bony surface on the tibial spacers. With the same pits of bony surface on the femoral spacers, increasing cement anchoring and decreasing loosening could be achieved; (4) filling all the depressions, defects, or gaps in the femoral and tibial insert models could allow smooth contours and decrease mechanical weakness in the cement spacer, avoiding cement crack or fracture.

The use of lubricant was necessary when fabricating COCM spacers with metallic mold [26], cement mold [7, 27], and polypropylene mold [24]. This led to multiple pitting or a rough joint surface, possibly increasing friction when gliding. Moreover, the cement mold had to be removed before the spacer cement cured; the articular surface of the spacer tends to be deformed, requiring reshaping. The cured CAD-articulating spacers were easily separated from the elastic silicon molds without changing the shape. Medical-grade silicone rubber TSE3488T (Momentive, Gunma, Japan) has low viscosity with excellent releasability, which meant that the lubricant was not necessary because the cement does not adhere to the interface of the silicone mold [28]. Moreover, the high tear and tensile strength of TSE3488T easily separated the cement spacer from the mold after the cement cured. This made a CAD-articulating spacer with perfect contour and ultra-high joint congruence. Moreover, these durable CAD trials and silicone molds are a highly cost-effective method for spacer fabrication because they can be easily cleaned, sterilized, and reused.

Struelens et al. [6] reported high mechanical complications (57%) with StageOne spacers, with more incidence of spacer tilting (24%) and mediolateral translation (21%). The cause might be related to poor fixation and poor constraint design of the spacers. However, Van Thiel et al. [25] recorded a complication of only 1.7% with the same spacers, which might be due to the postoperative restricted protocol of hinged knee brace or knee immobilizer for protection. Poor mechanical circumstances, such as bony loss, cruciate or collateral ligament defects following infection and resection arthroplasty, and poor constraining spacer design, made the infected knee joint inherently unstable and similar with the environment of revision TKA [23, 29]. This poor design of spacers resulted in more spacer-related mechanical complications, such as joint subluxation or dislocation [6]. Shen et al. [7] highlight that the tibial post can only maintain mediolateral stability, but the femoral cam is a key construct for anteroposterior stability and rollback mechanism. Most COCM spacers had no post-cam design [1, 19, 20] or had only a tibial post design [23, 24]. A post-cam design has been only seen in MOP spacers [5, 30, 31], Shen et al.’s spacers [7], and the CAD-articulating spacers in the present study. In comparison to primary TKA CS design, CAD-articulating spacers based on revision knee prosthesis had a larger box, cam, and post, which resulted in more constraint to counter the instability of the anteroposterior and mediolateral directions after infection. This could explain why knee braces were not necessary in our series.

The COCM spacers fabricated by Hsu et al. [24], Su et al. [23], and Shen et al. [7], and the COCH spacers produced by Villanueva-Martínez et al. [29], had acceptable complication rates ranging from 4.8% to 13.3%. The lower CAD-articulating spacer-related mechanical complication rate (6.3%) was similar to or lower than the rates found in previous studies. However, it was the first study to investigate spacer-related mechanical complications and to strictly define the sizing, position, and integrity of spacers and the relationship of bone and spacer of the femoral-tibial and patellar-femoral joints. Moreover, all the radiographs were reviewed independently by one musculoskeletal radiologist and two arthroplasty surgeons, which minimized the underestimation of the complication rate.

Ocguder et al. [22] used COCP spacer, which has International Organization for Standardization (ISO)-tested and ISO-approved mechanical properties and facilitates partial load-bearing. However, spacer cracks were detected in as high as 35.3%. The inclusion of high-dose antibiotics increases bactericidal capacity locally but also substantially weakens cement, especially hand-blended ones [32]. This might be the reason why antibiotic-loaded cement spacer cracks after even partial weight-bearing. Only toe weight-bearing was allowed in our series during the entire interim period; thus, no spacer crack or fracture occurred.

Haddad et al. [33] presented more complications with the earlier version of the PROSTALAC spacer (DePuy Synthes) due to minimal constraint design (non-posterior stabilized), but the complication rate deceased after those knees were managed with the current version of PROSTALAC spacer (posterior-stabilized). In their study, the total spacer-related mechanical complication rate (sum of two versions of PROSTALAC) based on our radiographic definition was 24.4%. In contrast with the results of Haddad et al., Meek et al. [31], and Gooding et al. [34] reported lower complication rates (2.1% and 6.1%, respectively) for the current PROSTALAC. With resterilized constrained MOP spacers, Hofmann et al. [30] and Pietsch et al. [35] reported lower mechanical complication rates (4.0% and 6.1%, respectively). There was 20.8% loosening of MOP spacers and 50.0% migration of COCH spacers in the series of Jämsen et al. [36], which might be related to the cement technique used, i.e., loose fixation of the spacer with cement. However, there was no spacer loosening or migration in our series, except in one medial plateau fracture with tibial spacer tilting, which was due to the patient’s dementia and full weight-bearing without protection. The multiple small pits over the bony surface of the CAD-articulating spacers could increase the roughness and allow better cement fixation. The metaphyseal stem-like cement could share the load of CAD-articulating spacers due to relatively poor bone stock after infection. The advantages of both mechanics could allow the spacer to be stably implanted in the bone, potentially minimizing the risk of spacer loosening or migration. Moreover, with the cementing technique we used [3], the spacers did not tightly interdigitate the bony surface, and the removal of spacers was simple with minimal bone loss. All the spacer-related mechanical complications of two-stage revision of periprosthetic knee infection that had been mentioned in the literature are summarized in Table 3.

**Table 3:** Summarizing the literature review of the spacer-related mechanical complications of two-stage revision of periprosthetic knee infection

Study	Spacer type	Numbers of spacers	Spacer-related mechanical complications	Complication rate (%)
Haddad et al. [33]	MOP	45	Subluxation in 5 Dislocation in 2 Extensor apparatus problems in 4	24.4
Meek et al. [31]	MOP	47	Spacer fracture in 1	2.1
Hofmann et al. [30]	MOP	50	Subluxation in 1 Extensor apparatus problems in 1	4.0
Pietsch et al. [35]	MOP	33	Dislocation in 1 Spacer migration in 1	6.1
Jämsen et al. [36]	MOP COCH	24 10	Spacer loosening in 5 Spacer migration in 5	20.8 50.0
Hsu et al. [24]	COCM	21	Subluxation in 1	4.8
Villanueva-Martínez et al. [29]	COCH	30	Spacer fracture in 1 Subluxation in 2	10.0
Su et al. [23]	COCM	15	Spacer migration in 2	13.3
Ocguder et al. [22]	COCP	17	Spacers cracks in 6	35.3
Shen et al. [7]	COCM	17	Spacer fracture in 1	5.9
Van Thiel et al. [25]	COCM	60	Spacer fracture in 1	1.7
Gooding et al. [34]	MOP	115	Spacer migration in 1 Spacer fracture in 3 Dislocation in 2 Extensor apparatus problems in 1	6.1
Struelens et al. [6]	COCM	155	Spacer tilting in 38 Spacer translation in 33 Spacer fracture in 7 Subluxation in 6 Dislocation in 4	57.0
Lin et al. (current study)	COCM	32	Spacer tilting in 1 Extensor apparatus problems in 1	6.3

*MOP* metal-on-polyethylene, *COCH* cement-on-cement handmade, *COCP* cement-on-cement prefabricated, *COCM* cement-on-cement molded

With multiple mechanical advantages, such as the corresponding geometry, matched sizing, balanced gap, ultra-high joint congruence, optimal post-cam constrain construction, and stable fixation, the optimized CAD-articulating spacer could significantly improve HSS knee score and ROM of the knee joint. With adequate debridement techniques and antibiotic-loaded spacers, our series shared a similar infection eradication rate (87.5%) with other studies [24, 27, 37, 38]. During reimplantation, there was no need for extensive release due to good soft tissue tension of the spacer. The ROM and HSS knee score after reimplantation significantly increased and improved, respectively. Increased ROM after reimplantation could be predicted from the good ROM at the interim stage [5, 39], as a theoretical functional outcome.

This study had some limitations. First, there were limited numbers of patients and the lack of a control group. Second, our case series only involved periprosthetic knee infection after primary TKA but did not include cases of post revision TKA. We did not have experience regarding whether CAD-articulating spacers could maintain stable joints if there were larger bony defects or more collateral ligament loss after TKA revision for infection. Third, nearly one third of the subjects underwent retention of the spacer because they were medically unfit. It was well reported that this situation was infrequent and had a high complication rate [40]. However, we did not record final functional score or ROM of these knees, but these spacers seemed to be positioned well and were stable, according to radiography at the outpatient clinic during the last follow-up. This might be ascribed to the novel congruent and more constrained design of CAD-articulating spacer. This study provides a platform for further evaluation of TKA two-step revision, similar to the study conducted by Choi et al. [41]. Lastly, we did not evaluate the biomechanical properties of CAD-articulating spacers like the experiment performed by Villa et al. [42]. However, if CAD-articulating spacers were tough enough to withstand failure on full weight-bearing, it would have been an added advantage. Elderly patients are likely to manage better with full weight-bearing while awaiting revision TKA. This provides another platform for further study.

## Conclusions

This was the first preliminary study to design and fabricate optimized CAD-articulating spacers based on retrieved knee prosthesis by reverse engineering and CAD/CAM techniques that provided multiple benefits. In two-stage revision of periprosthetic knee infection, the CAD-articulating spacer significantly controlled infection, improved clinical outcome, increased ROM, and decreased mechanical complications. Further larger clinical studies are needed to confirm the findings presented here.

## Abbreviations

3D: Three-dimensional
ANOVA: Analysis of variance
CAD: Computer-aided design
CAM: Computer-aided manufacture
COCH: Cement-on-cement handmade
COCM: Cement-on-cement molded
COCP: Cement-on-cement prefabricated
CR: Cruciate retaining
CRP: C-reactive protein
CS: Cruciate substituting
ESR: Erythrocyte sedimentation rate
HSS: Hospital of Special Surgery
ICC: Intra-class correlation coefficient
ISO: International Organization for Standardization
LCCK: Legacy Constrained Condylar Knee
MOP: Metal-on-polyethylene
MSIS: Musculoskeletal Infection Society
ROM: Range of motion
TKA: Total knee arthroplasty
